# Effect of Coronary Disease Characteristics on Prognostic Relevance of Residual Ischemia After Stent Implantation

**DOI:** 10.3389/fcvm.2021.696756

**Published:** 2021-12-07

**Authors:** Seokhun Yang, Jinlong Zhang, Doyeon Hwang, Joo Myung Lee, Chang-Wook Nam, Eun-Seok Shin, Joon-Hyung Doh, Masahiro Hoshino, Rikuta Hamaya, Yoshihisa Kanaji, Tadashi Murai, Jun-Jie Zhang, Fei Ye, Xiaobo Li, Zhen Ge, Shao-Liang Chen, Tsunekazu Kakuta, Bon-Kwon Koo

**Affiliations:** ^1^Department of Internal Medicine and Cardiovascular Center, Seoul National University Hospital, Seoul, South Korea; ^2^Department of Cardiology, The Second Affiliated Hospital Zhejiang University School of Medicine, Hangzhou, China; ^3^Division of Cardiology, Department of Internal Medicine, Heart Vascular Stroke Institute, Samsung Medical Center, Sungkyunkwan University School of Medicine, Seoul, South Korea; ^4^Department of Medicine, Keimyung University Dongsan Medical Center, Daegu, South Korea; ^5^Division of Cardiology, Ulsan Hospital, Ulsan, South Korea; ^6^Department of Medicine, Inje University Ilsan Paik Hospital, Goyang, South Korea; ^7^Division of Cardiovascular Medicine, Tsuchiura Kyodo General Hospital, Ibaraki, Japan; ^8^Division of Cardiology, Nanjing First Hospital, Nanjing Medical University, Nanjing, China

**Keywords:** coronary artery disease, atherosclerosis, fractional flow reserve, percutaneous coronary intervention, residual ischemia

## Abstract

**Objectives:** We investigated the influence of coronary disease characteristics on prognostic implications of residual ischemia after coronary stent implantation.

**Methods:** This study included 1,476 patients with drug-eluting stent implantation and available pre- and post-percutaneous coronary intervention (PCI) fractional flow reserve (FFR) measurements. Residual ischemia was defined as post-PCI FFR ≤ 0.80. Coronary disease characteristics with significant interaction hazard ratios (HRs) for clinical outcomes with residual ischemia were defined as interaction characteristics with residual ischemia (ICwRI). The primary outcome was target vessel failure (TVF)—a composite of cardiac death, target vessel myocardial infarction, and target vessel revascularization—at 2 years.

**Results:** The mean pre- and post-PCI FFR were 0.68 ± 0.11 and 0.87 ± 0.07, respectively. During the median follow-up duration of 2.0 years, the cumulative incidence of TVF was 6.1%. The 203 vessels (13.8%) with residual ischemia had higher risks of TVF compared to that for post-PCI FFR >0.80 (*P* < 0.001). ICwRI with a significant interaction HR with residual ischemia included pre-PCI SYNTAX score >17 and pre-PCI FFR ≤ 0.62. Each ICwRI had a direct prognostic effect not mediated by residual ischemia. The association between an increased TVF risk and residual ischemia was significant in patients with 0 or 1 ICwRI [hazard ratio (HR) 3.25, 95% confidence interval (CI) 1.90–5.57, *P* < 0.001] but not in those with 2 ICwRI (HR 0.47, 95% CI 0.14–1.64, *P* = 0.24). Among patients with post-PCI FFR >0.80, those with 2 ICwRI showed similar TVF risks to those with residual ischemia (HR 1.55, 95% CI 0.79–3.02, *P* = 0.20).

**Conclusions:** Coronary disease characteristics including pre-PCI SYNTAX score and pre-PCI FFR affected the prognostic implications of residual ischemia. The prognostic relevance of residual ischemia was attenuated in patients with multiple interacting characteristics.

## Introduction

Myocardial ischemia is a major prognostic determinant in patients with coronary artery disease (CAD) (1). Percutaneous coronary intervention (PCI) for patients with myocardial ischemia, defined by invasive physiologic indices such as fractional flow reserve (FFR) or non-hyperemic pressure ratios (NHPR), provides favorable outcomes compared to those for medical treatment alone (2–5). Nevertheless, PCI for lesions with low FFR or NHPR does not always mean optimal PCI, as up to 25% of patients succumb to residual ischemia after angiographically successful PCI (6, 7). Thus, post-PCI physiologic assessment is helpful in assessing the residual physiologic disease burden and the necessity for additional procedures.

Several studies have shown that the residual disease burden, as assessed by physiologic indices measured after PCI, is a prognostic indicator after coronary stent implantation (8, 9). However, the proposed cutoff values for physiologically optimal PCI varied and their sensitivity and specificity to predict clinical events were relatively low (10, 11). These findings limit the value of post-PCI physiological assessment as a guide for PCI optimisation and necessitate further investigations on the disease characteristics influencing the prognostic relevance of post-PCI physiologic assessment and the clinical benefit of additional PCI. Therefore, we investigated the influence of coronary disease characteristics on the clinical relevance of residual ischemia as well as their prognostic implications after coronary stent implantation.

## Materials and Methods

### Study Population

Patients from the International Post-PCI FFR registry (NCT04012281) who underwent second-generation drug-eluting stent (DES) implantation with pre-PCI FFR ≤ 0.80 and post-PCI FFR measurements were included. The registry is a patient-level pooled registry from four international studies (the COE-PERSPECTIVE registry, 3V-FFR-FRIENDS study, DKCRUSH-VII study, and the institutional registry at Tsuchiura Kyodo General Hospital) (8). Among these patients, 1,476 with available quantitative coronary angiographic (QCA) analysis and SYNTAX score were included in the present study (Supplementary Figure 1). The study protocol was approved by the institutional review boards of each participating center and in accordance with the principles of the Declaration of Helsinki.

### PCI and Pre- and Post-PCI Physiologic Assessments

Invasive coronary angiography was performed according to a standard protocol. Each core laboratory of four registries performed QCA analysis using a validated software program. DES type, stenting technique, number of stents, and adjunctive procedure were determined at the operating physician's discretion. Pre- and post-PCI FFR measurements were performed according to standard techniques using a pressure-temperature sensor guide wire (Volcano, San Diego, CA, USA) or St. Jude Medical (St. Paul, MN, USA). Continuous intravenous infusion of adenosine was used for hyperemia in 90.8% of patients, while intracoronary administration of papaverine, nicorandil, or adenosine was used in 9.2% of patients.

### Definitions and Clinical Outcomes of Residual Ischemia After PCI

Residual ischemia was defined as post-PCI FFR ≤ 0.80. The patients were divided into the residual ischemia and no residual ischemia groups. All analyses were performed using a per-patient analysis. In patients with multivessel disease, the vessel with the lowest post-PCI FFR was designated as a representative vessel. The primary outcome was target vessel failure (TVF), a composite of cardiac death, target vessel myocardial infarction (MI), and clinically driven target vessel revascularization (TVR) at 2 years. All deaths without an indicated non-cardiac cause were considered cardiac in nature. All definitions of clinical outcomes were based on those from the Academic Research Consortium (12).

### Coronary Disease Characteristics

The coronary disease or stenosis characteristics included left anterior descending artery (LAD) lesions, pre-PCI angiographic % diameter stenosis, minimum lumen diameter (MLD), reference diameter, lesion length, pre-PCI SYNTAX score, pre-PCI FFR, post-stent % diameter stenosis, post-stent MLD, residual SYNTAX score, % SYNTAX score decrease [i.e., (pre-PCI SYNTAX score—residual SYNTAX score)/pre-PCI SYNTAX score × 100], and % FFR increase [i.e., (post-PCI FFR—pre-PCI FFR)/pre-PCI FFR × 100]. Coronary disease characteristics with significant interaction hazard ratios (HRs) with residual ischemia (*P* < 0.10) were defined as interaction characteristics with residual ischemia (ICwRI) that could affect the clinical relevance of residual ischemia. Then, ICwRI was converted into binary variables based on the 75th percentile values. As a sensitivity analysis, the optimal cut-off value derived from Youden's index of receiver operating characteristic curve analysis was used for defining ICwRI.

### Statistical Analysis

Continuous and categorical variables are presented as means ± standard deviation and numbers (percentages), respectively. Paired *t*-tests were used to compare the distributions of pre- and post-PCI FFR. Cumulative event rates were evaluated using Kaplan–Meier censoring estimates. Cox proportional hazard regression was used to estimate the HRs and corresponding 95% confidence intervals (CIs). The HRs and 95% CIs of residual ischemia and post-PCI FFR were calculated in patients stratified by the presence of each ICwRI and the number of ICwRI. Mediation analysis was used to investigate the dependent and independent effects of ICwRI with residual ischemia as a mediator, TVF as an outcome, and each ICwRI as a treatment. Logistic regression was used to evaluate the association between treatment, mediator, and outcome. General algorithms were used to assess causal medication effects (13). All probability values were two-sided and *p* < 0.05 was considered statistically significant. Statistical analysis was performed using R version 3.6.2 (R Foundation for Statistical Computing, Vienna, Austria).

## Results

### Patient and Coronary Disease Characteristics

**Figure 1:**
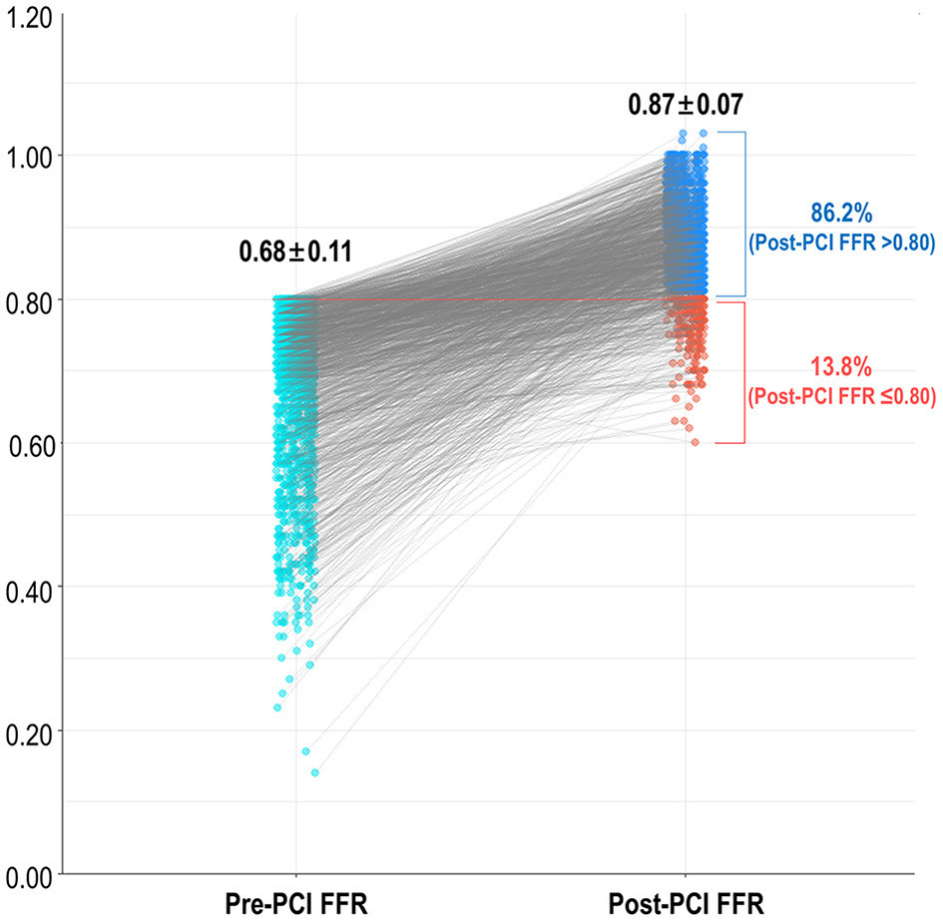
Frequency of residual ischemia after FFR-guided revascularization. FFR, fractional flow reserve; PCI, percutaneous coronary intervention.

The baseline patient and coronary disease characteristics of the study population are shown in Table 1. The mean % diameter stenosis, lesion length, and pre-PCI SYNTAX score were 62.6 ± 13.7%, 24.5 ± 14.1 mm, and 12.8 ± 7.3, respectively. The mean post-stent % diameter stenosis and residual SYNTAX score were 10.0 ± 7.0% and 3.7 ± 5.4, respectively. The mean pre-and post-PCI FFR were 0.68 ± 0.11 and 0.87 ± 0.07, respectively (*P* < 0.001). A total of 203 vessels (13.8%) had residual ischemia (post-PCI FFR ≤ 0.80) after revascularization (Figure 1).

**Table 1:** Baseline characteristics.

	**Total** **(*n* = 1,476)**
Clinical characteristics	
Age, years	63.5 ± 9.9
Male	1,150 (77.9)
Diabetes mellitus	479 (32.5)
Hypertension	969 (65.7)
Hypercholesterolemia	720 (48.8)
Smoking	423 (28.7)
Left ventricular ejection fraction, %	61.9 ± 8.4
Acute coronary syndrome	730 (49.5)
Previous myocardial infarction	193 (13.1)
**Coronary disease characteristics**	
Before PCI	
Location	
Left anterior descending coronary artery	1,117 (75.7)
Left circumflex artery	138 (9.3)
Right coronary artery	221 (15.0)
% Diameter stenosis	62.6 ± 13.7
Lesion length, mm	24.5 ± 14.1
Reference diameter, mm	2.86 ± 0.51
MLD, mm	1.06 ± 0.42
SYNTAX score	12.8 ± 7.3
FFR	0.68 ± 0.11
After PCI	
Post-stent % diameter stenosis	10.0 ± 7.0
Post-stent MLD, mm	2.72 ± 0.47
Residual SYNTAX score	3.7 ± 5.4
Post-PCI FFR	0.87 ± 0.07
Post-PCI FFR ≤ 0.80	203 (13.8)

*Values are mean ± standard deviation or n (%)*.

*FFR, fractional flow reserve; MLD, minimum lumen diameter; PCI, percutaneous coronary intervention*.

### Interactions Between Residual Ischemia and Coronary Disease Characteristics

**Figure 2:**
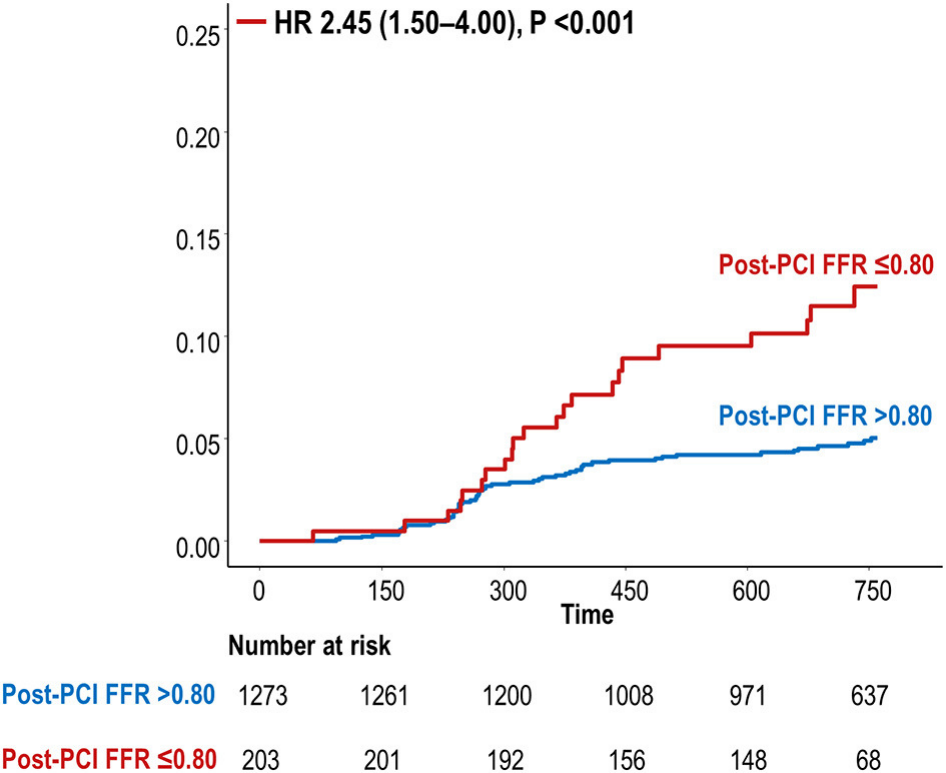
Cumulative incidence of TVF at 2 years according to residual ischemia. FFR, fractional flow reserve; HR, hazard ratio; PCI, percutaneous coronary intervention; TVF, target vessel failure.

During the median follow-up duration of 2.0 years, a total of 80 TVF events occurred (Supplementary Table 1). Patients with residual ischemia had a significantly higher risk of TVF at 2 years than that in patients without residual ischemia (HR 2.45, 95% CI 1.50–4.00, *P* < 0.001, Figure 2). Table 2 describes the interactions between coronary disease characteristics and residual ischemia for the prediction of TVF. Pre-PCI SYNTAX score and pre-PCI FFR showed an interaction with the prognostic impact of residual ischemia (p-for-interaction 0.042 and 0.073, respectively). Then, dichotomized variables based on 75th percentile value (i.e., pre-PCI SYNTAX score >17 and pre-PCI FFR ≤ 0.62) were defined as ICwRI.

**Table 2:** Coronary disease characteristics affecting the clinical relevance of residual ischemia.

	**Interaction HR** **(95% CI)**	***P*-value**
Pre-PCI angiographic characteristics		
Post-PCI FFR ≤ 0.80 * LAD lesion	1.59 (0.19–13.51)	0.672
Post-PCI FFR ≤ 0.80 * % Diameter stenosis	0.97 (0.93–1.01)	0.106
Post-PCI FFR ≤ 0.80 * MLD	1.46 (0.41–5.24)	0.558
Post-PCI FFR ≤ 0.80 * Reference diameter	0.51 (0.18–1.47)	0.212
Post-PCI FFR ≤ 0.80 * Lesion length	0.98 (0.95–1.02)	0.274
Post-PCI FFR ≤ 0.80 * pre-PCI SYNTAX score	0.94 (0.87–1.00)	0.042
Post-PCI FFR ≤ 0.80 * FFR	1.56 (0.96–2.55)	0.073
Post-PCI angiographic characteristics		
Post-PCI FFR ≤ 0.80 * Post-stent % diameter stenosis	1.05 (0.62–3.62)	0.163
Post-PCI FFR ≤ 0.80 * Post-stent MLD	0.76 (0.25–2.26)	0.617
Post-PCI FFR ≤ 0.80 * Residual SYNTAX score	1.05 (0.95–1.15)	0.338
Changes in angiographic characteristics		
Post-PCI FFR ≤ 0.80 * % SYNTAX decrease	0.99 (0.98–1.01)	0.536
Post-PCI FFR ≤ 0.80 * % FFR increase	0.98 (0.96–1.01)	0.187

*CI, confidence interval; FFR, fractional flow reserve; HR, hazard ratio; LAD, left anterior descending coronary artery; MLD, minimal lumen diameter; PCI, percutaneous coronary intervention*.

**Figure 3:**
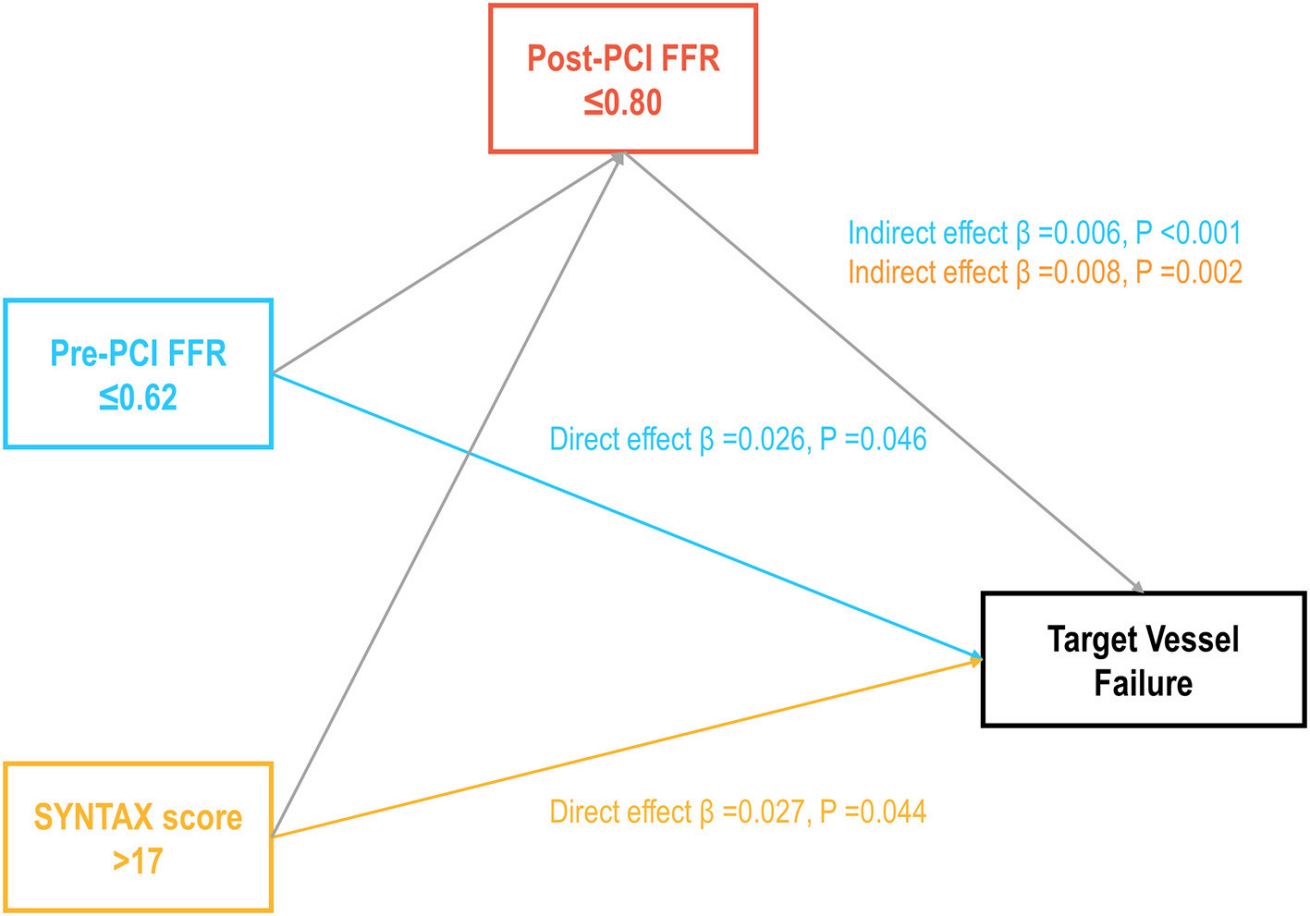
Prognostic implications of ICwRI mediated by residual ischemia. The direct and indirect prognostic effects of ICwRI mediated by residual ischemia are shown. ICwRI included pre-PCI SYNTAX score >17 and pre-PCI FFR ≤ 0.62. FFR, fractional flow reserve; ICwRI, interaction characteristics with residual ischemia; PCI, percutaneous coronary intervention.

Each ICwRI was associated with an increased risk of TVF at 2 years after stent implantation (HR 1.78, 95% CI 1.12–2.82, *P* = 0.014 for pre-PCI SYNTAX score >17; HR 1.65, 95% CI 1.05–2.61, *P* = 0.031 for pre-PCI FFR ≤ 0.62) and directly affected TVF not mediated by residual ischemia in a mediation analysis (Figure 3). The presence of residual ischemia was associated with an increased risk of TVF in patients with pre-PCI SYNTAX score ≤ 17 or pre-PCI FFR >0.62; however, its prognostic impact was attenuated in those with pre-PCI SYNTAX score >17 or pre-PCI FFR ≤ 0.62 (Table 3). This finding was consistent with those observed based on post-PCI FFR as a continuous variable (Table 3).

**Table 3:** Differential implications of residual ischemia and post-PCI FFR on clinical outcomes according to the ICwRI.

	**HR of post-PCI FFR ≤0.80** **(95% CI)**	***P*-value**	**HR of post-PCI FFR, per 0.1 increase** **(95% CI)**	***P*-value**
Pre-PCI SYNTAX score				
>17 (*N* = 351)	1.06 (0.45–2.48)	0.901	0.75 (0.45–1.23)	0.249
≤ 17 (*N* = 1,125)	3.58 (1.96–6.52)	<0.001	0.54 (0.37–0.78)	0.001
Pre-PCI FFR				
≤ 0.62 (*N* = 378)	1.01 (0.41–2.48)	0.984	0.71 (0.46–1.11)	0.132
>0.62 (*N* = 1,098)	3.77 (2.08–6.81)	<0.001	0.52 (0.35–0.77)	0.001

*ICwRI included pre-PCI SYNTAX score >17 and pre-PCI FFR ≤ 0.62*.

*CI, confidence interval; FFR, fractional flow reserve; HR, hazard ratio; ICwRI, interaction characteristics with residual ischemia; PCI, percutaneous coronary intervention*.

### Prognostic Impact of Residual Ischemia According to the Number of ICwRIs

**Figure 4:**
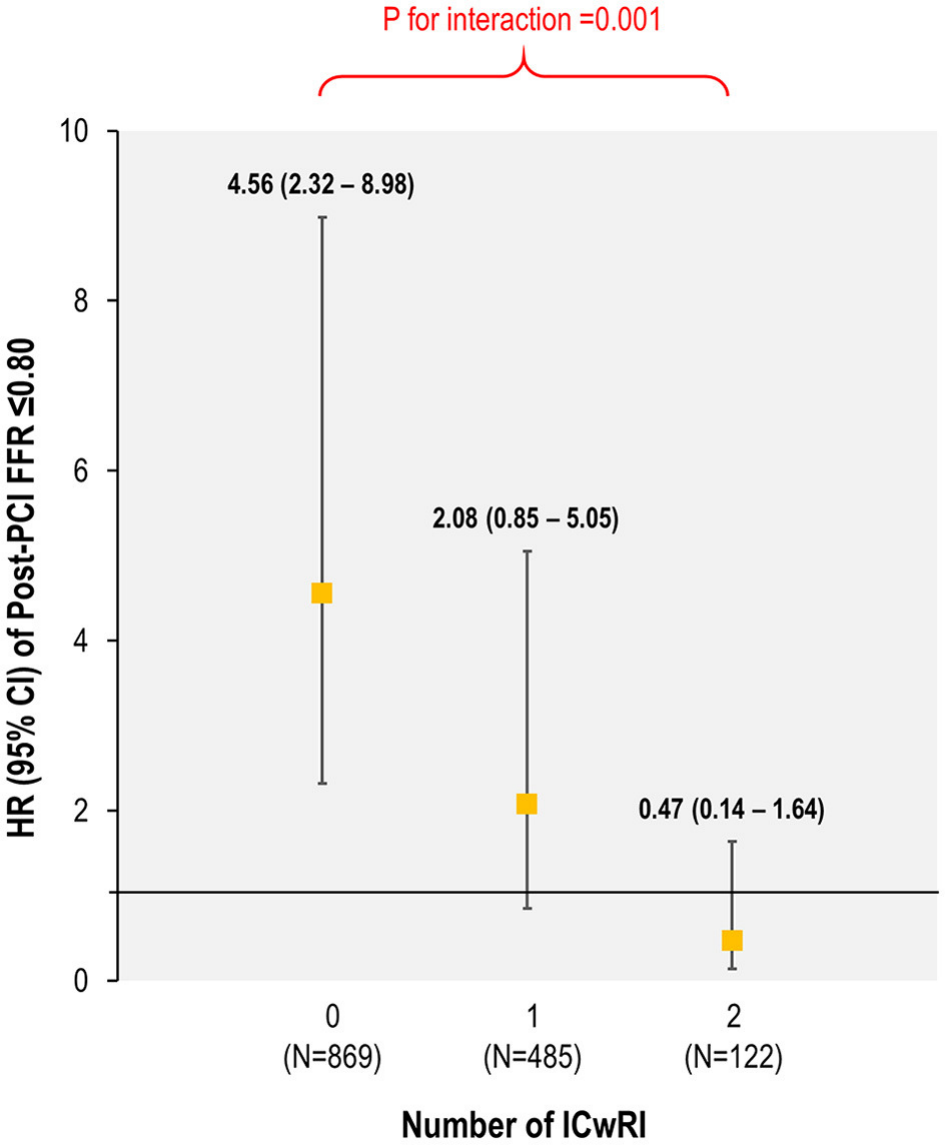
Differential prognostic implications of residual ischemia according to the number of ICwRI. The hazard for TVF at 2 years of residual ischemia according to the number of ICwRI is shown. The ICwRI definitions are described in Figure 3. CI, confidence interval; FFR, fractional flow reserve; HR, hazard ratio; ICwRI, interaction characteristics with residual ischemia; PCI, percutaneous coronary intervention.

The association between residual ischemia and TVF risk differed according to the number of ICwRI (*p*-for-interaction = 0.001); the prognostic impact of residual ischemia decreased as the number of ICwRI increased (Figure 4). The association between residual ischemia and TVF risk was significant in patients with 0 or 1 ICwRI (HR 3.25, 95% CI 1.90–5.57, *P* < 0.001) but not in those with 2 ICwRI (HR 0.47, 95% CI 0.14–1.64, *P* = 0.24).

**Figure 5:**
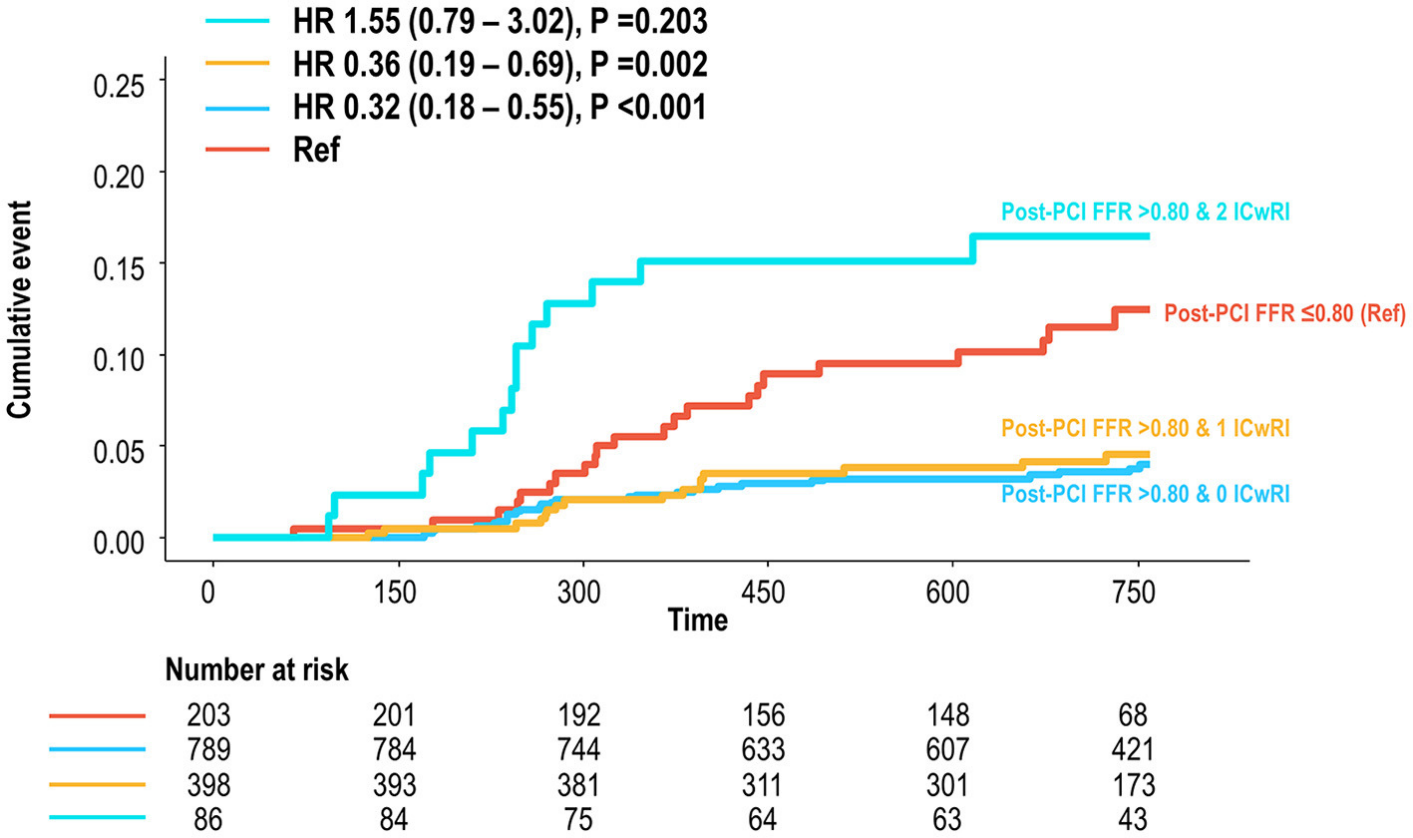
Cumulative incidence of TVF according to the number of ICwRI in patients without residual ischemia compared to those with residual ischemia. Patients with post-PCI FFR >0.80 were classified according to the number of ICwRI. The TVF risk for each group was then compared to that of patients with a post-PCI FFR of ≤ 0.80. The ICwRI definitions are shown in Figure 3. FFR, fractional flow reserve; HR, hazard ratio; ICwRI, interaction characteristics with residual ischemia; PCI, percutaneous coronary intervention; TVF, target vessel failure.

Comparisons of the outcomes of patients without residual ischemia (post-PCI FFR > 0.80) according to the number of ICwRI revealed a higher risk of TVF in patients with 2 ICwRI compared to that in patients with 0 or 1 ICwRI (HR 4.66, 95% CI 2.55–8.50, *P* < 0.001). The prognostic impact of 2 ICwRI in patients without residual ischemia was consistent in all subgroups with different clinical characteristics (Table 4). However, there was no significant difference in outcomes according to ICwRI in patients with residual ischemia (Supplementary Figure 2). Compared to the residual ischemia group, patients without residual ischemia and those with 0 or 1 ICwRI showed a significantly lower risk of TVF (HR 0.33, 95% CI 0.20–0.55, *P* < 0.001). However, those with 2 ICwRI had a similar risk of TVF as the residual ischemia group (HR 1.55, 95% CI 0.79–3.02, *P* = 0.203, Figure 5). In the sensitivity analysis, overall result was similar when the different cut-off values for pre-PCI SYNTAX score (>9.5) and pre-PCI FFR (≤ 0.66) were used to define ICwRI (Supplementary Figure 3).

**Table 4:** Prognostic implications of ICwRI among patients without residual ischemia according to their clinical characteristics.

	**HR of the number** **of ICwRI = 2** **(95% CI)**	***P*-value**	**Interaction** ***P*-value**
Age, years			0.161
≥65 (*N* = 634)	7.08 (3.27–15.30)	<0.001	
<65 (*N* = 639)	2.92 (1.10–7.74)	0.031	
Sex			0.210
Male (*N* = 981)	5.68 (3.00–10.74)	<0.001	
Female (*N* = 292)	1.42 (0.18–11.19)	0.741	
Diabetes mellitus			0.174
Yes (*N* = 401)	2.36 (0.70–7.98)	0.167	
No (*N* = 872)	6.34 (3.12–12.88)	<0.001	
Hypertension			0.033
Yes (*N* =841)	3.04 (1.41–6.53)	0.004	
No (*N* = 432)	12.37 (4.29–35.67)	<0.001	
Hypercholesterolemia			0.187
Yes (*N* = 608)	1.97 (0.46–8.48)	0.364	
No (*N* = 665)	5.76 (2.91–11.42)	<0.001	
Acute coronary syndrome			0.441
Yes (*N* = 654)	4.93 (2.49–9.77)	<0.001	
No (*N* = 619)	2.63 (0.61–11.33)	0.195	
Smoking			0.175
Yes (*N* = 348)	9.98 (2.92–34.11)	<0.001	
No (*N* = 925)	3.73 (1.85–7.49)	<0.001	
Previous myocardial infarction			0.272
Yes (*N* = 156)	1.62 (0.20–12.99)	0.647	
No (*N* = 1,117)	5.41 (2.87–10.19)	<0.001	
Left ventricular ejection fraction			0.997
≤ 40% (*N* = 31)	NA	NA	
>40% (*N* = 1071)	3.87 (1.93–7.78)	<0.001	

*The definitions of ICwRI were as in Table 3*.

*CI, confidence interval; FFR, fractional flow reserve; HR, hazard ratio; ICwRI, interaction characteristics with residual ischemia; PCI, percutaneous coronary intervention*.

## Discussion

This study investigated the influence of coronary disease characteristics on the prognostic implications of residual ischemia after coronary stent implantation. The main findings were as follows: (1) residual ischemia after PCI (post-PCI FFR ≤ 0.80) was associated with an increased risk of TVF at 2 years; the coronary disease characteristics affecting the prognostic relevance of residual ischemia, namely interaction characteristics with residual ischemia (ICwRI), were pre-PCI SYNTAX score >17 and pre-PCI FFR ≤ 0.62. Each ICwRI was a predictor of TVF, with a prognostic effect not mediated by residual ischemia. (2) The association between residual ischemia and TVF risk differed according to the number of ICwRI, and the prognostic impact of residual ischemia decreased as the number of ICwRI increased. (3) In patients with post-PCI FFR >0.80, those with 2 ICwRI showed a higher risk than those with 0 or 1 ICwRI, and those with post-PCI FFR of >0.80 and 2 ICwRI had a similar TVF risk with patients with residual ischemia.

### Clinical Relevance of Physiologic Assessment After PCI

Post-PCI physiologic assessment has been highlighted as an emerging tool to estimate the final result of coronary revascularization (14). Post-PCI FFR or NHPR is a surrogate marker of residual disease burden in both stented and non-stent segments after PCI (15–17) and demonstrates a risk continuum, with an inverse relationship with the risk of clinical events (8, 9). In the current study, patients with residual ischemia (defined as post-PCI FFR ≤ 0.80) showed a 2.5-fold increased risk of TVF compared to that in patients without residual ischemia. This finding is consistent with accumulated clinical evidence of the prognostic value of post-PCI FFR (14, 18). Although the prognostic value of post-PCI physiologic assessment has been established, the role of post-PCI physiologic indices as a procedural endpoint has been debated. Jeremias et al. reported that the proportion of residual disease in non-stent segments was about twice that than in stented segments among patients with impaired post-PCI NHPR due to untreated focal stenosis (7). Agarwal et al. reported subsequent intervention could reduce residual ischemia after PCI from 21 to 8% (19). However, Piroth et al. showed a low positive likelihood ratio of post-PCI FFR in the prediction of clinical outcomes from the randomized FAME (Fractional Flow Reserve or Angiography for Multivessel Evaluation) 1 and FAME 2 trials (10). Therefore, further investigation of the coronary disease characteristics determining the clinical relevance of residual ischemia or low post-PCI FFR or NHPR is needed for better application of post-PCI physiologic assessment in catheterization laboratories.

### Coronary Disease Characteristics Discriminative of the Prognostic Implications of Residual Ischemia

Various coronary disease characteristics are associated with adverse cardiovascular events after revascularization. However, the characteristics affecting the prognostic implications of residual ischemia have rarely been investigated. In the current study, pre-PCI SYNTAX score >17 and pre-PCI FFR ≤ 0.62 discriminated the prognostic relevance of residual ischemia. Moreover, mediation analysis showed that these ICwRIs were predictors of TVF at 2 years, independent of residual ischemia. Our observations are supported by previous findings. The baseline SYNTAX score was proportionally correlated with the incidence of 3-year composite outcomes after complete revascularization (20), and multivessel and diffuse diseases were associated with a >1.5-fold higher risk of clinical events after adjusting for a final FFR of ≤ 0.86 (19). Recently, an independent association between pre-PCI FFR and worse outcomes not mediated by post-PCI FFR was described (21). Although the independent prognostic role of pre-PCI FFR is still controversial and needs to be further elucidated in future studies (6, 22), these findings suggest that the risk of clinical events after PCI does not rely on a single attribute; rather, it is determined by complex interactions of baseline and residual anatomical and physiological disease burden. Interestingly, ICwRI did not encompass post-PCI angiographic parameters or relative changes in SYNTAX score or FFR in the current study. This finding is in accordance with worse outcome predictability of residual anatomical disease burden alone than residual anatomical and physiologic residual burden (23), and previous findings that post-PCI angiographic parameters were not predictive of post-PCI physiologic status (7).

### Clinical Application of the Concept of ICwRI

Several studies have proposed the clinical utilization of post-PCI physiologic assessment to optimize PCI results (7, 14, 19). Nonetheless, which patients should be paid more attention to obtain optimal post-PCI physiologic status have rarely been studied. The results of the current study demonstrated the differential prognostic impact of residual ischemia according to the number of ICwRIs. The association of residual ischemia with an increased risk of TVF at 2 years progressively increased with a decreasing number of ICwRI [HR 4.56 (2.32–8.98), HR 2.08 (0.85–5.05), and HR 0.47 (0.14–1.64) for patients with 0, 1, and 2 ICwRI, respectively]. This trend was driven by an increased risk of TVF in patients with a post-PCI FFR of >0.80 and 2 ICwRI, a similar risk to those with residual ischemia. Our finding of the attenuated association between residual ischemia and worse outcome according to ICwRI supports the previous report of the low likelihood ratio of post-PCI physiologic assessment (10). Although the exact mechanism of the interactive effect of ICwRI with the clinical relevance of residual ischemia requires elucidation, the baseline disease burden could be the main mediator to explain our observation. Pre-PCI SYNTAX score and pre-PCI FFR commonly reflect the anatomical and physiological atherosclerotic burden of a vessel and patient (24–27). Thus, patients with 0 or 1 ICwRI tend to have relatively low or moderate disease burdens, while patients with 2 ICwRI are likely to have a high disease burden. Although PCI is a procedure used to restore coronary blood flow (28) or seal local lesions to prevent acute events (29), it cannot fully reverse the risk of disease progression rooted in the coronary vasculature with high disease burden. Our findings indicate that ischemia relief by PCI or additional stent implantation to achieve optimal physiologic results could be preferred in patients with relatively low or moderate disease burden. Therefore, the clinical application of the ICwRI concept may be helpful in treatment planning and risk/benefit assessment of additional PCI in patients with low post-PCI FFR.

### Limitations

This study has several limitations. First, the study population was derived from observational registries that might have caused a lack of generalizability and causal relationship; thus, the results require further validation. Second, intravascular imaging was not performed in this study. Third, the cut-off value used for coronary disease characteristics in this study may not apply to other populations. Fourth, the hard outcomes could not be analyzed separately because of the low number of events. Fifth, the follow-up period was limited to 2 years and long-term follow-up data are needed to validate our findings.

## Conclusion

In this study, residual ischemia was associated with worse outcomes after PCI. However, the prognostic implication of residual ischemia diminished in patients with multiple coronary disease characteristics reflective of baseline disease burden, including pre-PCI SYNTAX score and pre-PCI FFR. Therefore, the integration of these coronary disease characteristics and post-PCI physiologic assessment will further refine post-PCI risk prediction and help appropriate treatment planning.

## Data Availability Statement

The datasets presented in this article are not readily available because data cannot be shared publicly due to the privacy of individuals that participated in the study. The data will be shared on reasonable request to the corresponding author. Requests to access the datasets should be directed to Bon-Kwon Koo, bkkoo@snu.ac.kr.

## Ethics Statement

The studies involving human participants were reviewed and approved by Seoul National University Hospital. Written informed consent for participation was not required for this study in accordance with the national legislation and the institutional requirements.

## Author Contributions

SY and JZ: conception, design, analysis, interpretation of data, drafting, revising of manuscript, and final approval of the manuscript submitted. DH, JL, C-WN, E-SS, J-HD, MH, RH, YK, TM, J-JZ, FY, XL, ZG, and S-LC: interpretation of data, revising of manuscript, and final approval of the manuscript submitted. TK and B-KK: conception, design, analysis, interpretation of data, drafting and revising of manuscript, and final approval of the manuscript submitted. All authors contributed to the article and approved the submitted version.

## Conflict of Interest

JL received a Research Grant from St. Jude Medical (Abbott Vascular) and Philips Volcano. J-HD received a Research Grant from Philips Volcano. B-KK received an Institutional Research Grant from St. Jude Medical (Abbott Vascular) and Philips Volcano. S-LC received a grant from the National Natural Scientific Foundation of China. The remaining authors declare that the research was conducted in the absence of any commercial or financial relationships that could be construed as a potential conflict of interest.

## Publisher's Note

All claims expressed in this article are solely those of the authors and do not necessarily represent those of their affiliated organizations, or those of the publisher, the editors and the reviewers. Any product that may be evaluated in this article, or claim that may be made by its manufacturer, is not guaranteed or endorsed by the publisher.
