# Supplementary material for: Effect of Coronary Disease Characteristics on Prognostic Relevance of Residual Ischemia After Stent Implantation
Source: Front Cardiovasc Med. 2021 Dec 7;8:696756. doi: 10.3389/fcvm.2021.696756 (PMC8688402; doi:10.3389/fcvm.2021.696756)
Supplement: Supplementary file 1 [file Data_Sheet_1.docx]

**Supplementary Materials**

**Effect of Coronary Disease Characteristics on Prognostic Relevance of Residual Ischemia After Stent Implantation**

**Supplementary Tables**

**Supplementary Figure Legends**

**Supplementary Table 1. Cumulative incidence and the number of clinical events**

| **Target vessel failure** | 6.1% (80) |
| --- | --- |
| Target vessel myocardial infarction | 0.5% (6) |
| Clinically driven target vessel revascularization | 5.2% (69) |
| Cardiac death | 0.9% (11) |

Cumulative incidence (number) of clinical events was presented as Kaplan-Meier estimates.

**Supplementary Figure Legends**

**Supplementary Figure 1. Study flow**

FFR, fractional flow reserve; PCI, percutaneous coronary intervention; QCA, quantitative coronary angiography.

**Supplementary Figure 2. Cumulative incidence of TVF according to ICwRI in patients with residual ischemia**

ICwRI included pre-PCI SYNTAX score >17 and pre-PCI FFR ≤0.62.

FFR, fractional flow reserve; HR, hazard ratio; ICwRI, interaction characteristics with residual ischemia; PCI, percutaneous coronary intervention.

**Supplementary Figure 3. Sensitivity analysis using optimal cut-offs for pre-PCI FFR and SYNTAX score**

In this sensitivity analysis, ICwRI was defined as pre-PCI SYNTAX score >9.5 and pre-PCI FFR ≤0.66 based on the optimal cut-off.

CI, confidence interval; FFR, fractional flow reserve; HR, hazard ratio; ICwRI, interaction characteristics with residual ischemia; PCI, percutaneous coronary intervention.
