# Supplementary figures and images for: Effect of Coronary Disease Characteristics on Prognostic Relevance of Residual Ischemia After Stent Implantation
Source: Front Cardiovasc Med. 2021 Dec 7;8:696756. doi: 10.3389/fcvm.2021.696756 (PMC8688402; doi:10.3389/fcvm.2021.696756)

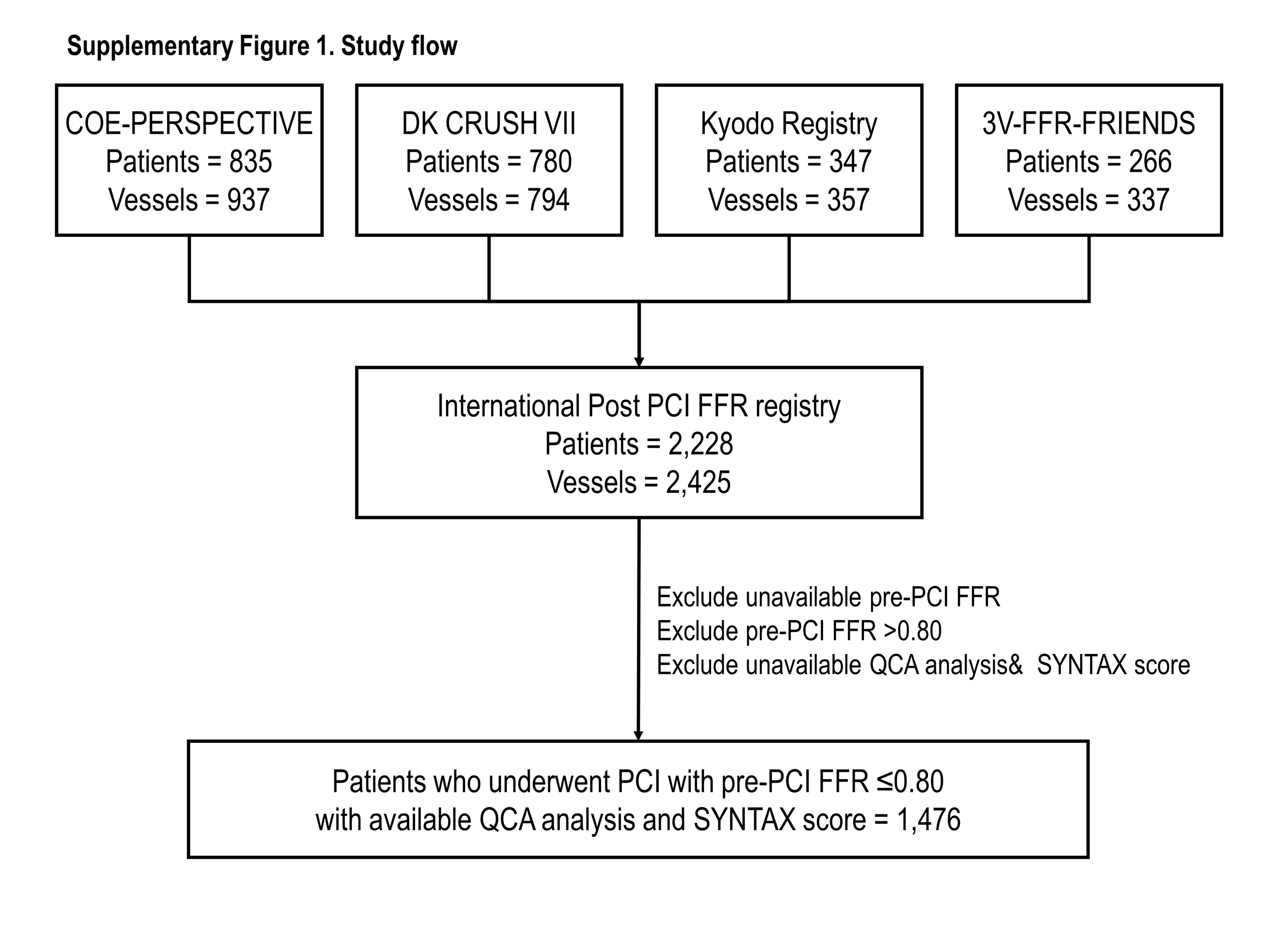

Supplement: Supplementary file 2 [file Image_1.TIF]

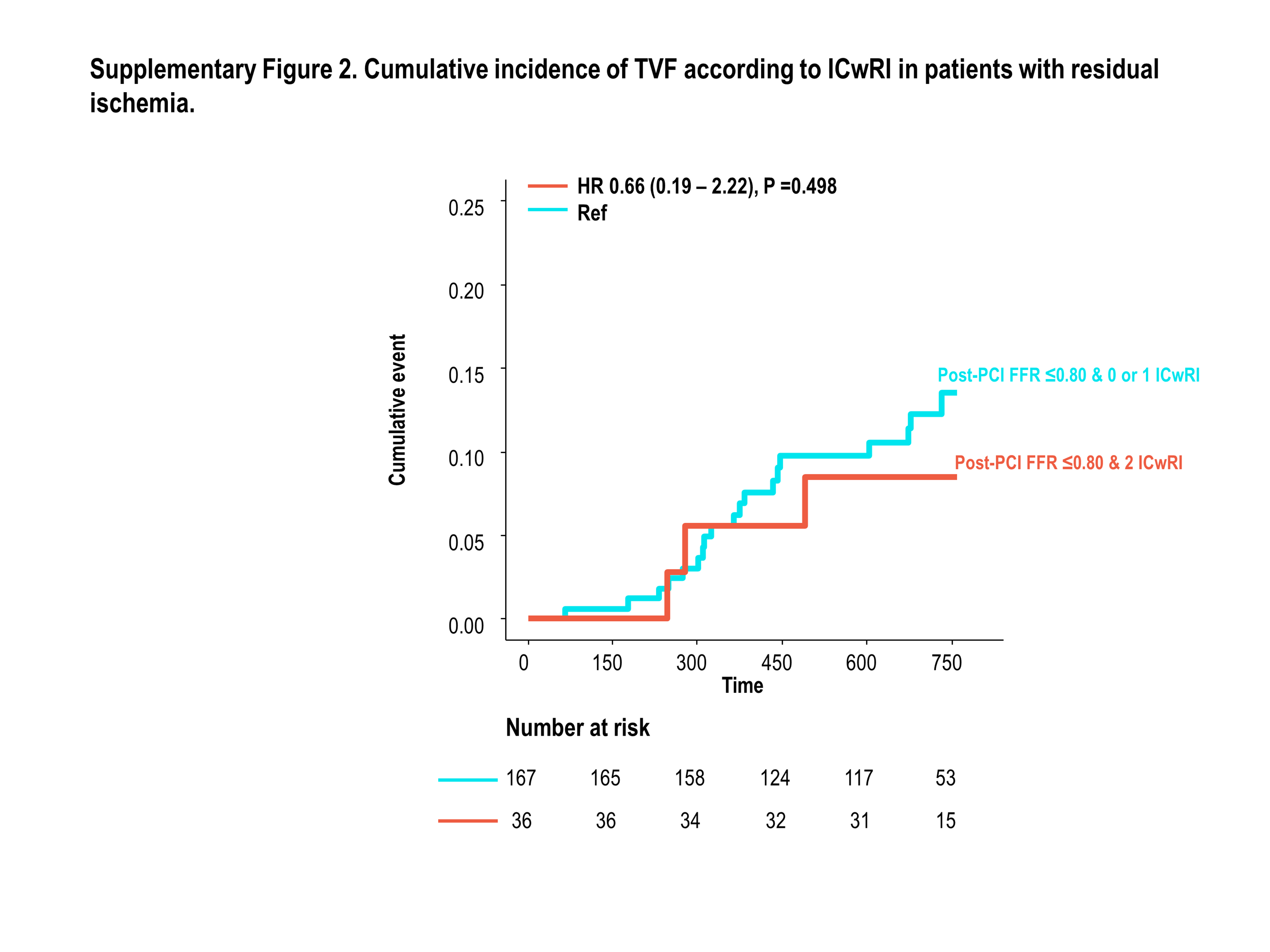

Supplement: Supplementary file 3 [file Image_2.TIF]

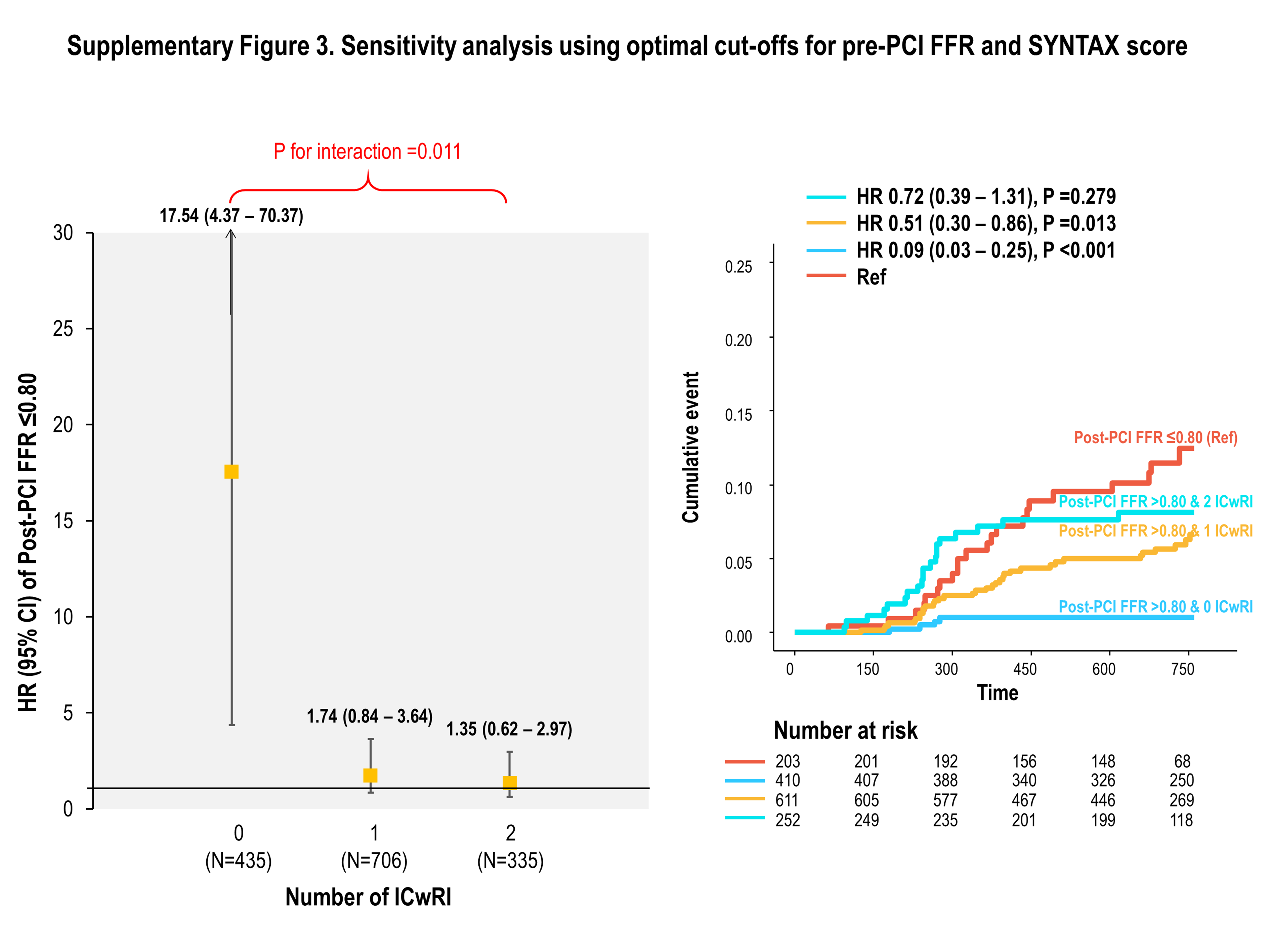

Supplement: Supplementary file 4 [file Image_3.TIF]
